# Differential insecticide resistance in *Anopheles arabiensis* populations in the seaside area of Mbour and its suburbs in Senegal

**DOI:** 10.1016/j.heliyon.2023.e21968

**Published:** 2023-11-06

**Authors:** Penda Sabaly, El Hadji Malick Ngom, Ndeye Astou Gueye, Assiyatou Gueye, Mawlouth Diallo, Ibrahima Dia

**Affiliations:** Pole de Zoologie Médicale, Institut Pasteur de Dakar, 36 Avenue Pasteur, BP, 220, Dakar, Senegal

**Keywords:** Molecular resistance, Monitoring, Malaria vectors, Mutations, Senegal

## Abstract

Regular monitoring of insecticide resistance status is an important step in implementing appropriate and adapted insecticide-based strategies for vector control. In Senegal, Indoor Residual Spraying (IRS) and a national distribution campaign for long-lasting insecticide-treated net (LLIN) have been implemented since 2007 and 2009, respectively to prevent malaria transmission. To expand and ensure the sustainability of these strategies, we conducted a study on the status of insecticide resistance in malaria vectors in the seaside area of Mbour and its suburbs where no data were previously available.

*Anopheles* larvae were sampled from four study sites (two in both coastal and inland areas) and reared to adulthood in the insectarium. Non-blood-fed females aged 3–5 days were then tested for susceptibility to permethrin, deltamethrin, lambdacyhalothrin, bendiocarb and pirimiphos-methyl. PCR amplification was used to identify sibling species of the *An. gambiae* complex and genotyping for the presence of resistance knockdown (kdr) *L1014S*, *L1014F* and *Ace-1 G119S*.

*Anopheles arabiensis* was the only species present in the area. At all four sites, mosquitoes were resistant to deltamethrin, permethrin, and lambdacyhalothrin, and exhibited varying degrees of resistance to bendiocarb and pirimiphos-methyl. Overall, high levels of leucine-serine/phenylalanine substitutions at position 1014 (*L1014S*/*L1014F*) were observed, with frequencies ranging from 76.4 to 85.2 % for *L1014F*, and from 43.2 to 66.7 % for *L1014S*, compared to 8.1 to 28.3 for the *Ace-1 G119S* mutation.

These results indicate a high level of phenotypic and genotypic resistance to insecticides, which is alarming, as it could have a significant impact on the operational effectiveness of current vector control tools that rely on pyrethroids. However, in the case of bendiocarb and pirimiphos-methyl, while some level of tolerance was observed, their potential use requires regular monitoring to prevent operational failure, as their deployment could potentially lead to an increase in resistance to them.

## 1 Introduction

Despite progress in control, malaria remains endemic in almost the entire WHO African region, and the progress in control efforts seems to be slowing [1]. In Senegal, substantial progress has been made in reducing malaria incidence through various strategies. These include large-scale implementation of seasonal malaria chemoprevention targeting children under 10 years of age [2], the distribution of long-lasting insecticidal nets [3], indoor residual spraying interventions [4,5], the use of artemisinin-based combination therapy [6], and mass testing and treatment [7]. However, the country is still part of the endemic zone where malaria continues to pose a significant public health challenge, especially in the southern regions. Indeed, in some regions such as the north, central, and mid-western parts of the country, these successful control interventions have led to a substantial reduction in malaria transmission [8]. This reduction can be attributed to the implementation and use of insecticide-based strategies, which have resulted in significant reductions in *Anopheles* mosquito populations and malaria transmission or incidence levels [4,5]. However, despite these achievements, there has been an emergence and spread of insecticide resistance among malaria vectors in selected sites, necessitating the replacement of insecticides from 2007 to 2014 to prevent vector control failure [5]. The primary mechanisms involved in this resistance are the kdr (*L1014F* and *L1014S*) and *Ace-1* G119S mutations [[9], [10], [11], [12], [13]]. To expand and ensure the sustainability of the available strategies, it is essential to characterize the resistance of malaria vectors to commonly used insecticides. While such data are available in several areas of Senegal, including the western [13,14] and southern regions of the country [9,[11], [15]], where insecticide resistance is widespread, there is a lack of data in many areas, including the seaside area of Mbour and its suburbs despite, its demographic and economic importance. This area was included in an IRS program, targeting to so-called “hotspots” with higher malaria transmission rates than the national average [4]. Specifically, two consecutive rounds of targeted IRS interventions were implemented in 2013 and 2014 using pirimiphos-methyl. Although the results indicated effectiveness in reducing the risk of malaria transmission, no information is available on the status of insecticide resistance in malaria vectors. To fill this gap, we conducted a study on the distribution of vectors and their insecticide resistance status. The aim was to collect baseline data that will help in the adaptation of vector control tools in this area with important economic characteristics as it is one of the most popular touristic destinations in Senegal.

## 2 Material and methods

### 2.1 Study sites

**Fig. 1:**
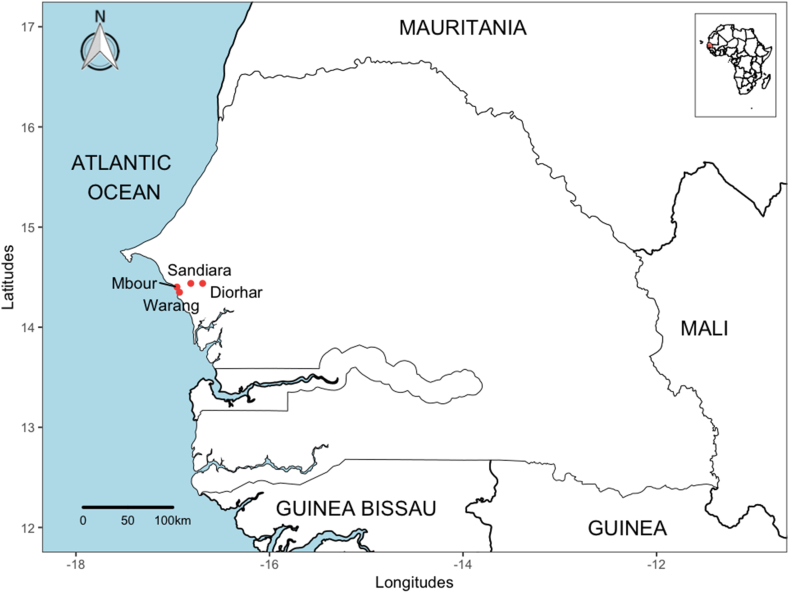
A map showing the sampling sites in Senegal.

The study was conducted in the Mbour region, situated along the west coast of Senegal, approximately 80 km south of Dakar and 5 km from the seaside resort of Saly Portudal, known for its various tourist attractions. The region experiences a typical Sudano-Sahelian climate characterized by a rainy season spanning from July to October, followed by a dry season from November to June. The annual average rainfall ranges from 500 to 800 mm and the daily temperatures vary between 18° and 31 °C. The population in this area exceeds 185,000 people, engaged in farming, pastoralism, or fishing activities. The primary agricultural crops cultivated include millet, peanuts, sorghum, maize, sesame, rice, beans, and various vegetables. The main domestic animals are cattle, sheep, goats, poultry and horses. The ethnic groups composition is diverse, with prominent groups including the Serer, Wolof, Pular, Bambara and Lebou. For the purpose of this study, four specific sites were selected based on human activities: Mbour and Warang, situated along the coastline and Diorhar and Sandiara, located in the interior (Fig. 1). Mbour is the most urbanized among the selected sites, while the others are categorized as semi-rural/urban areas.

### 2.2 Mosquito collection and rearing

Mosquitoes were collected in October 2019, which corresponds to the end of the rainy season. Larvae and pupae were collected from various breeding habitats, including semi-permanent and temporary pools, and subsequently reared to adulthood in the laboratory. They were maintained at 27 ± 2 °C and 75 ± 5 % relative humidity. Pupae were sorted and then transferred from rearing trays into beakers containing a small amount of water. These beakers were subsequently placed within an adult mosquito rearing cage, where the adult mosquitoes were provided with 10 % (w/v) sucrose solution to support their development.

### 2.3 Insecticide susceptibility tests

Insecticide susceptibility testing was conducted in accordance with the WHO procedure for such tests [16]. Non-blood-fed adult females, aged 3–5 days, were exposed to insecticide-impregnated papers containing discriminating concentrations of deltamethrin (0.05 %), permethrin (0.75 %), lambdacyhalothrin (0.05 %), bendiocarb (0.1 %), and pirimiphos-methyl (0.25 %). Batches of 20–27 mosquitoes, in four replicates were exposed to these insecticide-impregnated papers for a duration of 1 h in WHO test tubes. The number of mosquitoes that were knocked-down was recorded at 10, 15, 20, 30, 40, 50, and 60 min, allowing for the analysis of the dynamics of pyrethroids and the estimation the KD50 and KD95 times. For each insecticide, a control group (comprising 50 mosquitoes) was included in the testing, with these mosquitoes being simultaneously exposed to papers that were not impregnated with insecticide. After the test, the mosquitoes were transferred to holding tubes with untreated papers, where they were provided with a 10 % sucrose solution. Mortality among the mosquitoes was recorded 24 h after exposure. All surviving and dead specimens from the test were individually preserved in tubes containing silica gel for further molecular testing.

### 2.4 PCR assays

After morphological identification, genomic DNA was extracted from female *An. gambiae* s.l. mosquitoes using the CTAB protocol [17]. Identification at the species level was performed using intentional mismatch primers (IMPs), as detailed by Wilkins et al., [18]. Given their wide distribution in Senegal [[9], [10], [11], [12], [13]], the kdr mutations (*L1014F* and *L1014S*) and *Ace-1 G119S* mutation (*G119S*) were assessed using the primers described by Huynh et al. [19], and Weill et al. [20], respectively. For the PCR reaction, 2 μL of DNA template was combined with 23 μL PCR Master Mix containing *Taq* DNA Polymerase, RNAse/DNAse-free water, 5X Green GoTaq Buffer, 2.0–2.5 mM dNTP, 25 mM MgCl_2_ and 2.5–25 pmol/μL of specific primers. The PCR amplification was performed in an Eppendorf® Thermocycler with the following conditions: initial denaturation at 94°C–95 °C for 5 min for one cycle, followed by denaturation at 94°C–95 °C for 30 s, annealing at 58 °C (or 57/59 °C for *L1014S/L1014L* and 62 °C for *Ace-1 G119S*) for 30 s and, extension at 72 °C for 30 s, repeated for 30 cycles (or 35 cycles for *L1014S/L1014L* and *Ace-1 G119S*), and a final extension at 72 °C for 5 min. Afterward the *Ace-1 G119S* mutation amplicons were subjected to digestion with the restriction enzyme *Alu*I, and all amplicons were analyzed by migration on a 2.5 % agarose gel. Specific target bands were visualized under UV light.

### 2.5 Data analysis

After the susceptibility testing, when mosquito mortality in the control group fell within the range of 5 %–20 %, mortality was adjusted using the Abbott formula [21]. The determination of resistance status was based on WHO criteria as follows: 98–100 % mortality indicates susceptibility, 90–97 % mortality indicates tolerance or possible resistance (requiring further investigation), and mortality below 90 % indicates resistance. For pyrethroids, the times required to knock out 50 % and 95 % of mosquitoes (KD50 and KD95) were calculated using the R script developed by Karunarathne et al. [22], based on a log-probit model. The frequencies of the *L1014F, L1014S*, and *Ace-1 G119S* mutations were estimated for each site by the proportion in percentage between the number of individuals carrying each mutation over the total number of specimens tested. For the different exposure times, KD rates were estimated based on the number of mosquitoes knocked down out of the total number of specimens tested for pyrethroids. All analyses were performed using R 4.1.2 software [23].

## 3 Results

### 3.1 Insecticide susceptibility bioassays

A total of 2049 adult female F0 mosquitoes, aged 3–5 days and reared from larvae, were used to assess susceptibility to deltamethrin, lambdacyhalothrin, permethrin, bendiocarb and pirimiphos-methyl. In all the tests conducted, control groups showed no mortality, thereby the Abbott formula was not used. Table 1 presents the susceptibility status of the tested populations to the various insecticides used in this study. Resistance to pyrethroids was observed across all four sites, with recorded mortality rates for permethrin, deltamethrin, and lambdacyhalothrin below 90 %. Susceptibility to bendiocarb and pirimiphos-methyl was exclusively observed at the Warang site (Table 1).

**Table 1:** Mortality rates in *An. arabiensis* mosquitoes exposed to the different insecticides.

Insecticides	Sites	No. tested	Mortality (%)	Status	95%CI
Deltamethrin	Diorhar	102	60.8	Resistant	50.6–70.3
	Mbour	100	52	Resistant	41.8–62.1
	Sandiara	104	71.2	Resistant	61.5–79.6
	Warang	117	88.9	Resistant	81.8–94
Lambdacyhalothrin	Diorhar	102	53.9	Resistant	43.8–63.8
	Mbour	100	42	Resistant	31.2–52.3
	Sandiara	101	57.4	Resistant	43.8–63.8
	Warang	101	75.2	Resistant	65.7–83.3
Permethrin	Diorhar	100	57	Resistant	46.7–66.9
	Mbour	102	68.6	Resistant	58.7–77.5
	Sandiara	101	70.3	Resistant	60.4–79
	Warang	101	49.5	Resistant	39.4–59.6
Bendiocarb	Diorhar	102	93.1	Tolerant	86.4–97.2
	Mbour	102	87.3	Resistant	79.2–93
	Sandiara	103	96.1	Resistant	90.4–98.9
	Warang	100	98	Susceptible	93–99.8
Pirimiphos-methyl	Diorhar	101	90.1	Tolerant	82.5–95.2
	Mbour	97	72.2	Resistant	62.1–80.8
	Sandiara	100	78	Resistant	68.6–85.7
	Warang	114	99	Susceptible	95.2–100

CI: confidence interval.

### 3.2 Knock-down effects and dynamics of pyrethroids

**Fig. 2:**
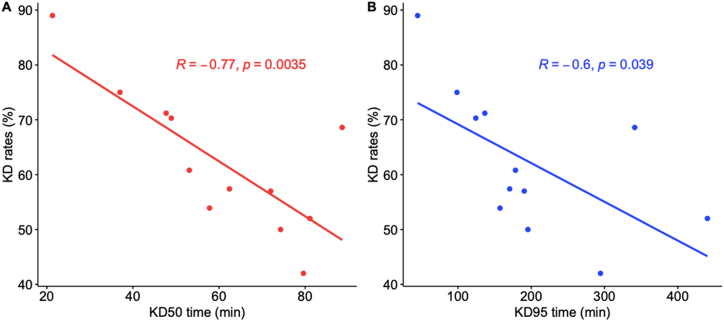
Correlation between KD50 and KD95 times and KD rates for the pyrethroids (**A**) KD50 and (**B**) KD95.

**Fig. 3:**
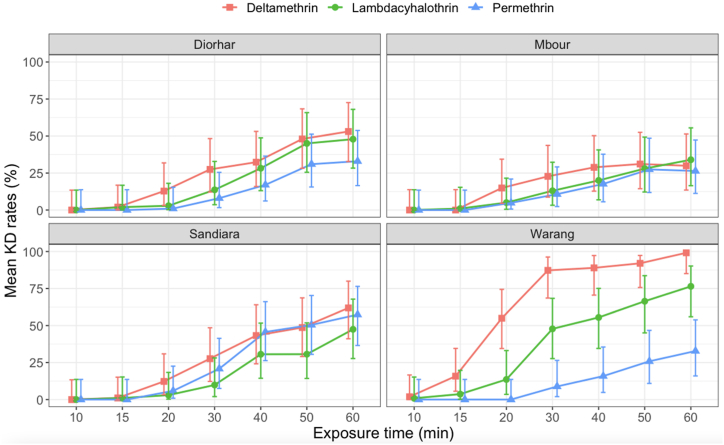
Variations of pyrethroids KD rates according to the exposure time.

Overall, the KD50 and KD95 times exhibited a significant negative correlation with KD rates levels for all three pyrethroids (Fig. 2A and B). For these pyrethroids, the KD levels were below 50 % throughout the 1-h exposure period in Mbour. A similar pattern was observed for permethrin and lambdacyhalothrin in Diorhar, lambdacyhalothrin in Sandiara and permethrin in Warang. The highest KD rates were recorded for deltamethrin and lambdacyhalothrin at the Warang site (88 % and 76 %, respectively), and for deltamethrin and permethrin (62.5 % and 57.4 %, respectively) at Sandiara, all after the 1-h exposure period (Fig. 3).

### 3.3 Species composition and distribution of L1014F, L1014S and G119S Ace-1 G119S mutations

**Fig. 4:**
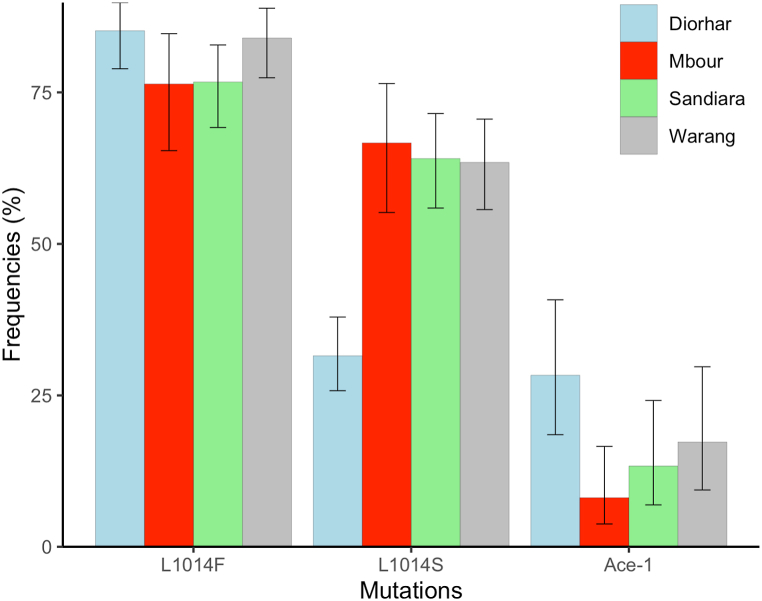
Variations of the frequencies of *L1014F*, *L1014S* and *Ace-1 G119S* mutations in the four sites. The bars indicate the upper and lower limits of the 95 % confidence intervals associated with the frequencies.

A total of 1398 adult *An. gambiae* s.l. mosquitoes were identified by PCR (372 from Diorhar, 371 from Mbour, 303 from Sandiara and 352 from Warang). All of these mosquitoes were identified as *An. arabiensis*. A total of 268, 266 and 123 specimens were genotyped for the presence of West kdr (*L1014F*), East kdr (*L1014S*) and *Ace-1 G119S* mutations. These mutations were detected at all four sites (Fig. 4). In the coastal sites, the *L1014F* mutation was recorded with allelic frequencies of 76.4 % in Mbour and 84 % in Warang. Conversely, in the inland sites, the frequencies were 85.2 % in Diorhar and 76.7 % in Sandiara. As for the *L1014S* mutation, frequencies were lower, ranging from 43.2 % in Diorhar and 66.7 in Mbour. In Warang and Sandiara, the frequencies were 63.5 % and 64.1 %, respectively. The lowest frequencies were observed for the *Ace-1 G119S* mutation, with rates of 8.1 % in Mbour, 17.3 % in Warang, 28.3 % in Diorhar, and 13.3 % in Sandiara. While there were no significant differences observed in the frequencies of *L1014F* (Chi-square test, χ^2^ = 4.7, *df* = 3, *P* = 0.20), the frequencies of *L1014S* and *Ace-1 G119S* mutations exhibited significant variation among the four sites (Chi-square test, χ^2^ = 20.8, *df* = 3, *P* = 0.0001 for *L1014S* and Chi-square test, χ^2^ = 15, *df* = 3, *P* = 0.002 for *Ace-1 G119S*).

## 4 Discussion

The discovery that *An. arabiensis* is the only species of the *An. gambiae* complex present in the Mbour region is in line with recent findings, as it accounted for approximately 99 % of the *Anopheles* specimens collected near Mbour [13]. This species has become well-established and is nearly the dominant species in nearby areas sharing similar ecological characteristics [10,[23], [24]]. Insecticide susceptibility testing revealed resistance to pyrethroids in all four sites, as evidenced by low mortality rates. This resistance was further supported by the results of KD effects. Furthermore, the relationship between KD rates and KD times exhibited linearity; with a more pronounced increase in KD time observed with permethrin compared to deltamethrin and lambdacyhalothrin. Several factors may contribute to this resistance, including selection pressure due to the increased use of LLINs in the vector control programs or for agricultural purposes, especially in Sandiara and Diorhar, as well as in Mbour, which is more urbanized. Additionally, recent IRS interventions [4] could have played a role in driving resistance. Across all four sampling sites, both the *L1014F* and *L1014S* kdr mutations were identified alongside the *Ace-1 G119S* mutation. Notably, the *L1014F* were more prevalent, consistent with previous observations in Senegal [25] and other regions of Africa [26,27]. However, discordant results have been observed in the urban area of Dakar [10] and in the central-western area of Senegal [14]. The degree of resistance conferred by these kdr mutations varied among study sites and depending on the specific insecticide tested. For type II pyrethroids, protection levels were less pronounced in Diorhar and Mbour, with the lowest mortality rates observed for deltamethrin and lambdacyhalothrin compared to Warang. This discrepancy may be attributed to higher selection pressure at the former sites, which are exposed to agrochemicals, as observed elsewhere [28,29]. In contrast, at Warang, where fishing is the primary activity, mortalities to deltamethrin and lambdacyhalothrin were higher than to permethrin, suggesting that type II pyrethroids may be more effective than type I [30,31]. This also indicates the likely presence of other resistance mechanisms that should be investigated. In addition to the presence of the kdr mutations, the detection of the *Ace-1 G119S* mutation, even at lower frequencies than the two mutations, raises concerns about the potential use of carbamates and organophosphates as alternatives. Indeed, these insecticides have been primarily used for IRS in the management of pyrethroid resistance in Senegal [5,32]. Given that susceptible or tolerant situations were observed in some areas, the observed resistance may have been recently selected following the extensive use of pirimiphos-methyl for IRS in Senegal and the study area [4,30].

While the *1014L*, *1014S* and *Ace-1 G119S* mutations have been identified, it is essential to investigate other mutations, such as *N1575Y*, which is involved in pyrethroid resistance. Moreover, biochemical monitoring using synergists and gene expression analysis should be investigated to gain a more comprehensive understanding of the various mechanisms involved. Future studies should focus on closely monitoring resistance to carbamates and organophosphates to prevent the failure of control strategy failures within these two chemical families. Additionally, the impact of agriculture selection pressure should be thoroughly assessed.

## Author contribution statement

Penda Sabaly, El Hadji Malick Ngom, Mawlouth Diallo and Ibrahima Dia: Conceived and designed the experiments. Penda Sabaly, El Hadji Malick Ngom, Ndeye Astou Gueye, Assiyatou Gueye and Ibrahima Dia: Performed the experiments; analyzed and interpreted the data. Ibrahima Dia and Mawlouth Diallo: Contributed reagents, materials, analysis tools or data, Penda Sabaly, El Hadji Malick Ngom, Ndeye Astou Gueye, Assiyatou Gueye, Mawlouth Diallo and Ibrahima Dia: Wrote the paper.

## Funding statement

This study was funded by 10.13039/501100003762Institut Pasteur de Dakar.

## Data availability statement

All available data are included in the article.

## Additional information

No additional information is available for this paper.

## Declaration of competing interest

The authors declare that they have no known competing financial interests or personal relationships that could have appeared to influence the work reported in this paper.

## References

[1] Dhiman S. (2019). Are malaria elimination efforts on right track? An analysis of gains achieved and challenges ahead. Infect Dis Poverty.

[2] Ndiaye J.L., Cissé B., Ba E.H., Gomis J.F., Ndour C.T., Molez J.F., Fall F.B., Sokhna C., Faye B., Kouevijdin E., Niane F.K., Cairns M., Trape J.F., Rogier C., Gaye O., Greenwood B.M., Milligan P.J.M. (2016). Safety of seasonal malaria chemoprevention (SMC) with sulfadoxine-pyrimethamine plus amodiaquine when delivered to children under 10 years of age by district health services in Senegal: results from a stepped-wedge cluster randomized trial. PLoS One.

[3] Thwing J.I., Perry R.T., Townes D.A., Diouf M.B., Ndiaye S., Thior M. (2011). Success of Senegal's first nationwide distribution of long-lasting insecticide-treated nets to children under five - contribution toward universal coverage. Malar. J..

[4] Sy O., Niang E.H.A., Ndiaye M., Konaté L., Diallo A., Ba E.C.C., Tairou F., Diouf E., Cissé B., Gaye O., Faye O. (2018). Entomological impact of indoor residual spraying with pirimiphos-methyl: a pilot study in an area of low malaria transmission in Senegal. Malar. J..

[5] Lo C., Dia A.K., Dia I., Niang E.H.A., Konaté L., Faye O. (2019). Evaluation of the residual efficacy of indoor residual spraying with bendiocarb (FICAM WP 80) in six health districts in Senegal. Malar. J..

[6] Thiam S., Thwing J., Diallo I., Fall F.B., Diouf M.B., Perry R., Ndiop M., Diouf M.L., Cisse M.M., Diaw M.M., Thior M. (2020). Scale-up of home-based management of malaria based on rapid diagnostic tests and artemisinin-based combination therapy in a resource-poor country: results in Senegal. Malar. J..

[7] Conner R.O., Dieye Y., Hainsworth M., Tall A., Cissé B., Faye F., Sy M.D., Ba A., Sene D., Ba S., Doucouré E., Thiam T., Diop M., Schneider K., Cissé M., Ba M., Earle D., Guinot P., Steketee R.W., Guinovart C. (2020). Mass testing and treatment for malaria followed by weekly fever screening, testing and treatment in northern Senegal: feasibility, cost and impact. Malar. J..

[8] NMCP (2019). https://pnlp.sn/wp-content/uploads/2020/11/Bulletin-Epidemiologique-ANNUEL-2019-du-Paludisme-au-SENEGAL-VFinale.pdf.

[9] Niang E.H.A., Konate L., Diallo M., Faye O., Dia I. (2016). Patterns of insecticide resistance and knock down resistance (kdr) in malaria vectors *An. arabiensis, An. coluzzii* and *An. gambiae* from sympatric areas in Senegal. Parasites Vectors.

[10] Dia A.K., Guèye O.K., Niang E.A., Diédhiou S.M., Sy M.D., Konaté A., Samb B., Diop A., Konaté L., Faye O. (2018). Insecticide resistance in *Anopheles arabiensis* populations from Dakar and its suburbs: role of target site and metabolic resistance mechanisms. Malar. J..

[11] Gueye O.K., Tchouakui M., Dia A.K., Faye M.B., Ahmed A.A., Wondji M.J., Nguiffo D.N., Mugenzi L.M.J., Tripet F., Konaté L., Diabate A., Dia I., Gaye O., Faye O., Niang E.H.A., Wondji C.S. (2020). Insecticide resistance profiling of *Anopheles coluzzii* and *Anopheles gambiae* populations in the southern Senegal: role of target sites and metabolic resistance mechanisms. Genes.

[12] Diallo M., Hamid-Adiamoh M., Sy O., Sarr P.C., Manneh J., Ndiath M.O., Gaye O., Faye O., Konaté L., Sesay A.K., Assogba B.S., Niang E.H.A. (2021). Evolution of the pyrethroids target-site resistance mechanisms in Senegal: early stage of the Vgsc-1014F and Vgsc-1014S Allelic frequencies shift. Genes.

[13] Sy O., Sarr P.C., Assogba B.S., Ndiaye M., Dia A.K., Ndiaye A., Nourdine M.A., Guèye O.K., Konaté L., Gaye O., Faye O., Niang E.A. (2021). Detection of kdr and ace-1 mutations in wild populations of *Anopheles arabiensis* and *An. melas* in a residual malaria transmission area of Senegal. Pestic. Biochem. Physiol..

[14] Thiaw O., Doucouré S., Sougoufara S., Bouganali C., Konaté L., Diagne N., Faye O., Sokhna C. (2018). Investigating insecticide resistance and knock-down resistance (kdr) mutation in Dielmo, Senegal, an area under long lasting insecticidal-treated nets universal coverage for 10 years. Malar. J..

[15] Diouf E.H., Niang E.H.A., Samb B., Diagne C.T., Diouf M., Konaté A., Dia I., Faye O., Konaté L. (2020). Multiple insecticide resistance target sites in adult field strains of *An. gambiae* s.l. from southern Senegal. Parasites Vectors.

[16] WHO (2018). http://www.who.int/malaria.

[17] Morlais I., Cohuet A., Ponçon N., Simard F., Fontenille D. (2004). Intraspecific nucleotide variation in *Anopheles gambiae*: new insights into the biology of malaria vectors. Am. J. Trop. Med. Hyg..

[18] Wilkins E.E., Howell P.I., Benedict M.Q. (2006). IMP PCR primers detect single nucleotide polymorphisms for *Anopheles gambiae* species identification, Mopti and Savanna rDNA types, and resistance to dieldrin in *Anopheles arabiensis*. Malar. J..

[19] Huynh L.Y., Sandve S.R., Hannan L.M., Ert M.V., Gimning J.E. (2007).

[20] Weill M., Malcolm C., Chandre F., Mogensen K., Berthomieu A., Marquine M., Raymond M. (2004). The unique mutation in Ace-1 giving high insecticide resistance is easily detectable in mosquito vectors. Insect Mol. Biol..

[21] Abbott W.S. (1925). A method of computing the effectiveness of an insecticide. J. Econ. Entomol..

[22] Karunarathne P., Pocquet N., Labbé P., Milesi P. (2022). BioRssay: an R package for analyses of bioassays and probit graphs. Parasites Vectors.

[23] R Core Team (2021). https://www.R-project.org/.

[24] Fontenille D., Lochouarn L., Diatta M., Sokhna C., Dia I., Diagne N., Lemasson J.J., Ba K., Tall A., Rogier C., Trape J.F. (1997). Four years entomological study of the transmission of seasonal malaria in Senegal and the bionomics of *Anopheles gambiae* and *An. arabiensis*. Trans. Roy. Soc. Trop. Med. Hyg..

[25] Ndiath M.O., Cailleau A., Orlandi-Pradines E., Bessell P., Pagès F., Trape J.-F., Rogier C. (2015). Emerging knock-down resistance in *Anopheles arabiensis* populations of Dakar, Senegal: first evidence of a high prevalence of kdr-e mutation in West African urban area. Malar. J..

[26] Stump A.D., Besansky N.J., Vulule J.M., Atieli F.K. (2004). Dynamics of the pyrethroid knockdown resistance allele in western Kenyan populations of *Anopheles gambiae* in response to insecticide-treated bed net trials. Am. J. Trop. Med. Hyg..

[27] Kawada H., Futami K., Komagata O., Kasai S., Tomita T., Sonye G., Mwatele C., Njenga S.M., Mwandawiro C., Minakawa N., Takagi M. (2011). Distribution of a knockdown resistance mutation (L1014S) in *Anopheles gambiae* s.s. and *Anopheles arabiensis* in western and southern Kenya. PLoS One.

[28] Gimonneau G., Bouyer J., Morand S., Besansky N.J., Diabate A., Simard F. (2010). A behavioral mechanism underlying ecological divergence in the malaria mosquito *Anopheles gambiae*. Behav. Ecol..

[29] Dadzie S., Appawu M.A., Kerah-Hinzoumbe C., Akogbeto M.C., Adimazoya M., Israel D.K., Fadel A.N., Williams J. (2016). Species composition and insecticide resistance status of *Anopheles gambiae* (s.l.) (Culicidae) in Kome, southern Chad and the implications for malaria control. Parasites Vectors.

[30] Reimer L., Fondjo E., Patchoké S., Diallo B., Lee Y., Ng A., Ndjemai H.M., Atangana J., Traore S.F., Lanzaro G., Cornel A.J. (2008). Relationship between kdr mutation and resistance to pyrethroid and DDT insecticides in natural populations of *Anopheles gambiae*. J. Med. Entomol..

[31] Burton M.J., Mellor I.R., Duce I.R., Davies T.G.E., Field L.M., Williamson M.S. (2011). Differential resistance of insect sodium channels with kdr mutations to deltamethrin, permethrin and DDT. Insect Biochem. Mol. Biol..

[32] Sy O., Niang E.H.A., Diallo A., Ndiaye A., Konaté L., Ba E.H.C.C., Tairou F., Cissé B., Gaye O., Milligan P., Faye O. (2019). Evaluation of the effectiveness of a targeted community-based IRS approach for malaria elimination in an area of low malaria transmission of the central-western Senegal. Parasite Epidemiol. Control.

